# Changes in diabetic and renal profile of people exposed to fluoride in south India

**DOI:** 10.6026/97320630018820

**Published:** 2022-09-30

**Authors:** Kurpad Nagaraj Shashidhar, Munilakshmi Uppalamethi, Sai Deepika Ram Mohan, Deena Mendez, Raveesha Anjanappa

**Affiliations:** 1 Sri Devaraj Urs Medical College, Kolar - 563103

**Keywords:** Type 2 diabetes mellitus, insulin sensitivity, insulin resistance, insulin, fluorosis

## Abstract

Type 2 Diabetes Mellitus is leading cause of Diabetic microvascular complications. India stands second across the globe in prevalence of diabetes mellitus. Due to deficit rain fall, the water table is exposed to more of salts and minerals from the rocks underground. One of the minerals is the Fluoride. Fluoride in negligible amount is good for dental health, chronic exposure to higher range of fluoride causes various metabolic disturbances. Aim: To study the effect of chronic fluoride exposure on diabetes mellitus. A total of 288 study subjects were recruited. The blood samples and urine samples were collected from all the study subjects. Study groups; Group1: Healthy Controls, Group2: Type 2 Diabetes Mellitus and Group3: Diabetic Nephropathy. The serum (0.313± 0.154) and urine (0.3±0.6) fluoride values of diabetic nephropathy group were significantly decreased in comparison between groups. The primary objective of the fluoride with insulin (-0.06) levels are inversely correlating and fluoride with microalbumin (0.083) levels are directly correlating. Results of the study gave a clear picture of effect of fluoride on insulin action and renal damage. In conclusion, though there is no significant effect of fluoride on FBS, PPBS and HbA1c, insulin is the determining factor for glucose homeostasis which is decreased. Microalbumin is yet another marker for renal clearance which is increased. Therefore, fluoride shall be considered as a parameter in prognosis of metabolic disorder especially Diabetes mellitus in fluoride endemic areas.

## Background:

Diabetes is a serious, chronic disease that occurs either when the pancreas does not produce enough insulin or when the body cannot effectively use it [1]. Prevalence of diabetes is gaining the status of a potential epidemic in India [1]. Indians are genetically predisposed to microvascular complications [2, 3]. Etiology of diabetes in India is multi factorial and includes genetic factors coupled with environmental influences [2, 4]. Kolar district a semi urban area located in Karnataka has recorded an exponential increase in the incidence of diabetes mellitus and its associated microvascular complications [5]. This increased incidence may be due to environmental factors and one of these factors may be fluoridated water [5]. Fluorine the most electronegative element of periodic table and is found as fluoride ion in nature (F-)[6]. Despite its benefits due to its anti-cariogenic activity, this ion can produce dental and/or skeletal fluorosis [6]. The deleterious effects of F are not limited to the calcified tissues but also poseseveral toxic effects on the endocrine system and soft tissues. These led to disturbances causing preeclampsia, disturbances in glucose homeostasis [7, 5]. Fluoride has been shown to increase blood glucose levels and impair glucose tolerance. Impaired glucose tolerance often is a precursor of type 2 diabetes and has been found to occur in humans with fluoride intake of 0.07- 0.4 mg/kg/day [8]. Studies have shown that chronic intake of high levels of fluoride leads to hyper glycaemia with high plasma insulin levels and inhibit glycolytic enzyme [9]. Studies have documented that insulin secretion decreases in presence of concentrations of fluoride higher than 5 µmol/L [9]. The molecular mechanisms involved would be related to the operation of the signaling systems involving cAMP, protein kinase C, and intracellular calcium [10]. These mechanisms and flaws made us to evaluate the effect of F on insulin secretion and to find if any association of diabetes and related microvascular complications of fluorosis in the local population.

##  Materials and Methods:

### Study Design:

Cross Sectional Study Total number of study subjects (n=288) 

Group I: Non- Diabetic no microvascular complications (n=96) 

Group II: Diabetes type 2 without microvascular complications (n=96)

Group III: Diabetes type 2 with nephropathy (n=96)

With confidence level = 95%; Margin of error = 10%; response distribution = 50 %

The diagnostic criterion for diabetes is fasting plasma glucose ≥ 126 mg/dl and post prandial plasma glucose ≥ 140 mg/dl [11]. Diagnostic point selected on the basis of micro-vascular complications such as diabetic retinopathy and nephropathy is based on urine albumin creatinine ratio (UACR ≥ 30 mg/g) [11]. UCAR is estimated in the spot urine.

### Study area:

Study was approved by SDUAHER Central Ethics Committee No: SDUAHER/KLR/R&D/45/2017- 18. All diabetic cases reported to RL Jalappa Hospital, the teaching Hospital of Sri Devaraj Urs Medical College, constituent of Sri Devaraj Urs Academy of Higher Education and Research, Kolar were selected. For the Subjects enrolled in the study Physiological variables like Systolic and Diastolic Blood Pressure were measured.

### Sample Collection:

All the study subjects are residents of Kolar district for a minimum period of three years. Informed written consent was obtained from all study subjects. Age and gender matched non diabetics and healthy subjects of same area were included as controls. Blood sample of 5 mL and urine sample of 10 mL was collected and stored under -20°C until analysis.

### Duration of the study:

2 years (2018- 2020)

### Variables:

Serum Creatinine, Serum Uric Acid, Serum Sodium, Potassium and Urine Micro albumin by Vitros 5, 1 FS (Dry Chemistry fully automated analyzer). Serum and urine Fluoride by Fluoride ISE by Thermo Scientific Fischer, USA, HbA1c by BioRad D10 and Plasma Insulin by Vitros eCI.

### Statistical analysis:

Samples analyzed were tabulated in the Microsoft Excel sheet and performed statistical analysis package of SPSS version 22.0 such as student t test, One Way ANOVA and Pearson's Correlation to find out the significance (p<0.05).

### Inclusion criteria:

[1] Subjects clinically proven with Type 2 DM

[2] Subjects clinically proven with diabetic nephropathy

[3] Subjects living in Kolar district for minimum period of 3years

### Exclusion criteria:

[1] Patients with diabetes mellitus not living in Kolar and not exposed to fluoride

[2] Patients taking drugs or other factors known to cause diabetes and/ or diabetic nephropathy

[3] Patients undergoing renal dialysis

[4] Acute kidney injury due to any cause and other renal pathologies

[5] Patients with other type of diabetes

## Results and Discussion:

Table 1(see PDF) tabulates the basic details of systolic and diastolic blood pressure, which is significantly increased in diabetic nephropathy (DN) group when compared with controls and T2DM. HbA1c was significantly increased in both the cases when compared with controls. Renal profile (Serum urea, creatinine and microalbumin) was significantly increased in DN group when compared with the other two groups. Serum fluoride and urine fluoride was significantly decreased in DN. On the other hand, Uric acid was decreased in T2DM group, but was significantly increased in DN group when compared to group 2.Correlation between essential variables was performed in groups 2 and 3 which are tabulated in Table 2(see PDF). Blood pressure and renal parameters were significantly and positively correlating in cases. However, Systolic blood pressure which is determining factor for renal insufficiency was significantly correlating with microalbumin only in DN group [12].Calculated parameters to assess insulin action were included and tabulated in Table 3(see PDF). Across the groups the Insulin resistance and insulin sensitivity was significantly increased and decreased respectively in cases. Correlation of Fluoride with insulin and microalbumin was performed in cases. DN cases were affected directly in insulin values and microalbumin concentration significantly. This is evident from Table 4(see PDF). To evaluate the fluorosis, basic demographic details of the subjects were recorded. Systolic and Diastolic Blood Pressure (BP) showed a marginal increase in type 2 Diabetes and diabetic nephropathy group (Groups 2 and 3). In group 3, patients with diabetic nephropathy, only the SBP was significantly high giving a clue that the SBP is the parameter which will be affected during renal injury which is accordance with the study conducted by Noshad et al.[12].Therefore, in blood pressure of patients especially SBP is still a marker for Diabetic nephropathy [10]. From Table 2(see PDF), there was no significant difference Systolic blood pressure (SBP) and Diastolic blood pressure (DBP) between groups 1 and 2. From Table 1(see PDF), FBS was significantly increased in all the diabetic groups when compared with controls (92.21±13.259). Similarly, PPBS of groups 2 (262.31±96.34) &3 (262.83±84.04) were equal and not significant indicating that the metabolism of glucose after a meal in all the cases are almost the same. Close monitoring of PPBS may prevent microvascular complications which were documented by S Fu et al for cardio vascular stiffness [13]. Glycated Hemoglobin (HbA1c) was significantly increased in all the cases when compared to the control group (5.540±.5003). In a study conducted by Jian-bin Su et al, increased HbA1c led to increased diabetic peripheral nephropathy which is one among the microvascular complication which implies the same in our study with respect to nephropathy [15]. Glucose metabolism mainly depends on hormones; insulin and glucagon. In our study we considered insulin to assess insulin resistance and sensitivity, fasting insulin was analyzed which was very high in DR cases compared to other groups. Insulin was significantly varied in group 3 (10.9±11) when compared with groups 1 (11.50±6.84) and 2 (14.92±12.90). This increase in turn may be compared with BMI giving a clue for insulin action on cell. Hyper insulinemia may lead to increased metabolic disturbances thereby aggravating the disorder [16]. Thus, comparison of diabetic profile with the demographic details there are few important novel outcomes affecting progression of Type 2 Diabetes Mellitus to micro vascular complications and finally to the end stage of the complications.

The cases recruited for DN group were not on dialysis however, they were in initial stages of the disorder. Type 2 Diabetic cases admitted in general medicine ward with increased serum creatinine and urea levels were reassessed for the renal profile to confirm the microvascular complication. The renal profile of DN group was increased compared to other groups confirming diabetic nephropathy [17]. Urea (84.12± 38.99), Creatinine (3.86± 2.239) and uric acid (5.94± 4.69) were increased in group 3. . Serum Fluoride was not significantly varied when compared between controls (0.633±0.183) and type 2 diabetes cases (0.665±0.29). The urinary fluoride concentration of all the groups were more than the serum fluoride indicating normal clearance of fluoride through renal system except in group 3 (0.3±0.6). The disproportionate serum and urine fluoride excretion is in nephropathy group may be due to renal damage leading to decreased urine fluoride values. Microalbumin was analyzed in the all the subjects. Microalbumin was sharply increased group 3 (532.22±549.6) indicating severe renal damage in initial stages also [16]. Therefore, a significant increase was observed between group 3 and other groups. In brief, estimation of these biochemical parameters gives a gist that in diabetic nephropathy fluoride clearance is hindered which gives an insight for further studies to evaluate the molecular damage in renal cells. From Table 2(see PDF), the correlation of SBP with microalbumin was not significant in type 2 DM cases (-0.124) though it was significantly correlating in groups 2 (0.198) and 3 (0.241) indicating that micro albumin can be considered as a marker of renal dysfunction a well as early marker of detection of renal dysfunction in retinopathy cases [16]. Since 2 groups are diabetic and also have increased glucose levels, the correlation of FBS and PPBS with HbA1c remains as significant positive correlation. In contrast, with microalbumin as biomarker of renal tissue damage, serum urea and creatinine are considered for initial evaluation of the state of kidney [17]. There are no much evidence stating the importance of correlation between parameters. From the present study correlation of urea and creatinine was significantly positive in all the three groups unlike micro albumin which was not significant in group 2 which proves that the trend of this duo is maintained since years as biomarkers of renal insufficiency and found better when compared to microalbumin and SBP .Insulin profile of the present study includes HOMA- IR and QUICKI tabulated in Table 3(see PDF). HOMA- IR. Increase in resistance decreases insulin reception by cell and thereby impaired glucose metabolism [18]. Increase in sensitivity increases reception of insulin by cells. From Table 3(see PDF) it is deemed fit to accept that control subjects have increased insulin sensitivity than other groups [19]. Therefore, insulin profile shall be considered for better understanding of glucose metabolism of an individual [18, 19]. The major objective of the study is to find the effect of fluoride on renal efficiency and insulin action. From Table 4(see PDF), it is clear that in Diabetic nephropathy, due to increased serum fluoride and decreased urine fluoride, fasting insulin is directly proportional and microalbumin is inversely proportional to serum fluoride levels [20]. These results indicate the renal damage is prevalent in fluoride endemic area as suggested by Deepika et al. [20].

## Conclusions:

In summation, after the diagnosis of type 2 diabetes mellitus, management and prognosis of the disorder is of prime importance. The environmental factors also play a major role wherein, fluorosis is given prominence in fluoride endemic area as in the present study. There was no notable effect of fluoride on the routine diabetic investigations such as fasting blood sugar, post prandial blood sugar and HbA1c. However, correlation of fluoride with Fasting insulin, HOMA-IR, QUICKI, and microalbumin must be envisaged for better management of diabetic microvascular complications. As a concluding remark, insulin sensitivity and resistance can act as surrogate marker in managing diabetes thereby preventing its complications. The same may also hold good to assess the impact of fluoride on the insulin which may act as basis for further molecular studies on fluorosis.
